# Morphological and molecular description of *Serpinema yehi* n. sp. (Nematoda: Camallanidae), parasite of *Rhinoclemmys punctularia* (Testudines: Geoemydidae) from Brazilian Amazon

**DOI:** 10.1017/S0031182026102509

**Published:** 2026-08

**Authors:** Leandro Mauricio Oliveira Silva, Lorena Freitas Souza Tavares-Costa, Ronald Ferreira Jesus, Marlon Leal, Jeannie Nascimento Santos, Francisco Tiago Vasconcelos Melo

**Affiliations:** 1 Institute of Biological Sciences, Federal University of Pará (UFPA), Belém, Brazil; 2 Vale Institute of Technology Sustainable Development, Belém, Brazil

**Keywords:** Brazilian amazon, camallanidae, phylogeny nematode, *Rhinoclemmys punctularia*, *Serpinema*

## Abstract

*Serpinema* currently comprises 12 valid species of nematode parasites of freshwater turtles, distributed across the Neotropical, Oriental and Palearctic regions. Thus, we describe *Serpinema yehi* n. sp., a parasite of *Rhinoclemmys punctularia* from the Brazilian Amazon. Species identification was based on light and scanning electron microscopy, and molecular and phylogenetic analyses were performed using partial sequences of the mitochondrial cytochrome c oxidase subunit I and nuclear 18S genes. *Serpinema yehi* n. sp. can be distinguished from its congeners by the number of ridges in the buccal capsule, the number and arrangement of caudal papillae, the length and morphology of the right spicule, vulvar lip morphology and the number of mucrons on the female tail. Phylogenetic analyses recovered the new species as closely related to *Camallanus*, but not nested within the *Camallanus* spp. clade, thereby supporting the recognition of *Serpinema* as a valid genus. *Serpinema yehi* n. sp. represents the second species of the genus described from *R. punctularia* and the eighth species recorded from Brazil.

## Graphical abstract

**Figure:**
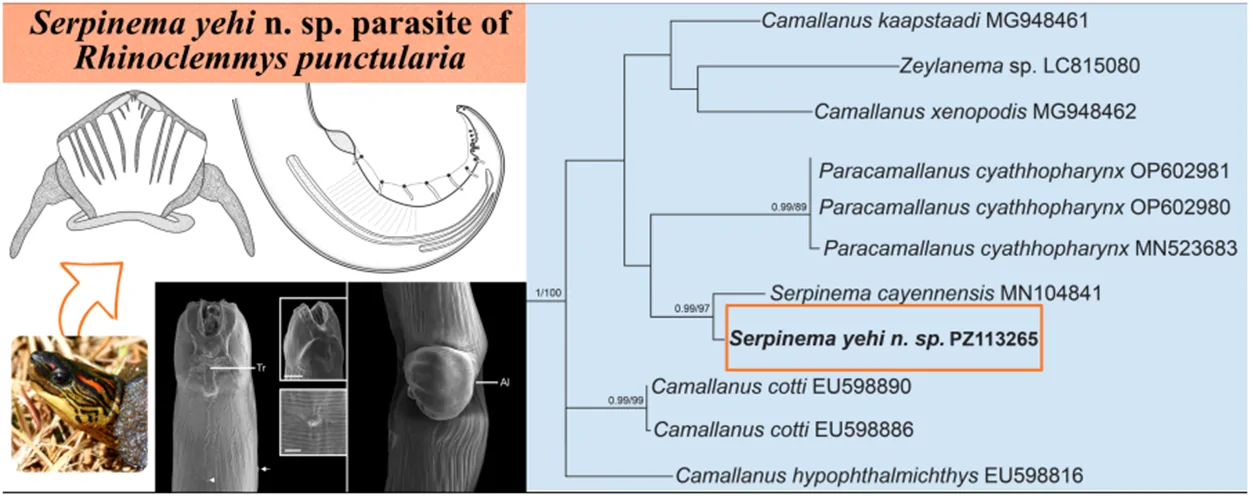

## Introduction

*Serpinema* Yeh, 1960 (Nematoda: Camallanidae) currently includes 12 valid species (Silva et al., 2023, 2026) whose adult stages parasitize the gastrointestinal tract of freshwater turtles and occurring in the Neotropical, Oriental and Palearctic regions (Ivashkin et al., 1977; Harnoster et al., 2019; Silva et al., 2023). Members of this genus are characterized by a well-sclerotized buccal capsule formed by two lateral, shell-like valves, with internal longitudinal ridges arranged into dorsal and ventral groups, separated by a distinct gap (Yeh, 1960; Silva et al., 2026).

*Serpinema* was erected based on 10 species of the genus *Camallanus* to found parasitizing freshwater turtles (Yeh, 1960), namely: *Se. amazonicum* (Ribeiro, 1941), *Se. intermedius* (Hsu and Hoeppli, 1931), *Se. kachugae* (Baylis and Daubney, 1922), *Se. magathi* (Sprehn, 1932), *Se. magnorugosus* (Caballero, 1939), *Se. microcephalus* (Dujardin, 1845), *Se. octorugatum* (Baylis, 1933), *Se. ptychozondis* (MacCallum, 1918), *Se. trispinosum* (Leydi, 1851) and *Se. undulatus* (Railliet and Henry, 1915). Subsequently, *Se. magnorugosus* was regarded as a junior synonym of *Se. trispinosum* (Moravec and Vargas-Vázquez, (1998)), whereas *Se. ptychozondis* and *Se. undulatus* were considered invalid (Sharma et al., 2002). In addition, *Se. lissemysus* (Gupta and Singh, 1959) was not included by Yeh (1960) and four further species were later described: *Se. cayennensis* Harnoster, Svitin and Du Preez, 2019, *Se. emydidium* (Mascarenhas and Müller, 2017), *Se. monospiculatum* Freitas and Dobbin Jr., 1962, and *Se. pelliculatum* Silva, Jesus and Melo, 2023 (Harnoster et al., 2019; Silva et al., 2026).

*Serpinema* spp. are morphologically very similar to those of the genera *Camallanus* Railliet and Henry, 1915 and *Zeylanema* Yeh, 1960, which has led several authors to question the validity of *Serpinema* and *Zeylanema* as independent taxa. Consequently, both have been treated either as synonyms (Stromberg and Crites, 1974; Rigby and Rigby, 2014) or as subgenera (Baker, 1983; Moravec and Scholz, 1991; Moravec and Van As, 2015) of *Camallanus*.

The taxonomic status of *Serpinema* was previously addressed by Harnoster et al., (2019), who conducted phylogenetic analyses of species of *Camallanus* and *Serpinema* using three molecular markers. Analyses of the 28S gene showed that, although *Serpinema* spp. did not form a fully distinct clade, *Se. octorugatum* and *Se. cayennensis* did not cluster with other *Camallanus* species, and the 18S and COI analyses indicated more distant relationships between *Se. cayennensis* and *Camallanus* spp. parasitizing fishes and amphibians.

Based on the evidence supporting the validity of the genus *Serpinema* provided by Harnoster et al., (2019) and Ailán-Choke and Pereira (2021), Silva et al. (2026) proposed an identification key for the genera of Camallanidae. In this key, *Serpinema* and *Zeylanema* are recognized as valid genera, and the authors emphasize that the current family classification does not support the recognition of subgenera.

In this context, seven species of *Serpinema* have been recorded in Brazil, 6 of which parasitize freshwater chelonians: *Se. amazonicum, Se emydidium, Se. magathi, Se. microcephalus, Se. monospiculatum* and *Se. pelliculatum*. Additionally, larval stages of *Se. trispinosum* have been reported to infect fish (Falkenberg et al., 2024; Silva et al., 2026).

During a helminthological survey of parasites of freshwater turtles, nematodes were recovered from the gastrointestinal tract of *Rhinoclemmys punctularia* (Daudin, 1801) in the Brazilian Amazon region. The specimens were assigned to the genus *Serpinema*, with morphological features that clearly differentiate them from their congeners. Thus, the present study aims to describe a new species of *Serpinema* based on morphological and molecular data.

## Materials and methods

### Nematode collection and morphological analysis

We examined the gastrointestinal tracts of four specimens of *Rhinoclemmys punctularia* collected in July 2022 in the municipality of Abaetetuba, Pará State, Brazil (SISBIO license no. 53527–4). Hosts were anaesthetized by injection of 2% ketamine and subsequently euthanized by ketamine overdose. All internal organs were removed and examined under a Leica EZ4 stereomicroscope (Leica Microsystems, Wetzlar, Germany). Nematodes were washed in 0.9% saline solution, fixed in heated 70% ethanol and preserved in microtubes with room-temperature 70% ethanol. Some specimens were stored at 4 °C in 100% ethanol for molecular analyses.

For morphological and morphometric analyses, specimens were cleared in Amann’s lactophenol, mounted on temporary slides and examined under an Olympus BX41 microscope (Olympus, Tokyo, Japan) equipped with a drawing tube. For scanning electron microscopy (SEM), 6 specimens (3 males and 3 females) were post-fixed in 1% OsO₄ (osmium tetroxide), dehydrated through an ascending graded ethanol series, dried at the CO₂ critical-point, mounted on metallic stubs, coated with gold–palladium (Silva et al., 2024) and analysed under a Vega3 microscope (TESCAN, Brno, Czech Republic) with an accelerating voltage of 10–20 kV, at the Laboratory of Structural Biology (LBE), Federal University of Pará (UFPA).

All measurements are given in micrometres unless otherwise stated and are presented as Holotype/allotype, followed by the mean value and minimum and maximum measurements of the type series indicated in parentheses. Prevalence, mean abundance and mean intensity were calculated using the methods of Bush et al., (1997). The specimens were deposited in the Other Invertebrate Collection of Museu Paraense Emílio Goeldi (MPEG), Pará, Brazil.

### Molecular and phylogenetic analyses

For molecular analyses, nematode specimens were preserved in microtubes with absolute ethanol and stored at −20 °C. Specimens used for DNA extraction were sectioned, and the anterior and posterior regions of a single female specimen were preserved and stored separately in ethanol. The mid-body region was used for DNA extraction. The hologenophore, as defined by Pleijel et al., (2008), was preserved and deposited as a voucher specimen in a helminthological reference collection (MPEG).

Genomic DNA was extracted using the DNeasy Blood and Tissue Kit (Qiagen) according to the manufacturer’s standard protocol, and we amplified the ribosomal 18S gene and the mitochondrial cytochrome c oxidase subunit I (COI) gene. The 18S gene was amplified using the primers 18S_F (forward) (5′-CGC GAA TRG CTC ATT ACA ACA GC-3′) and 18S_R (reverse) (5′-GGG CGG TAT CTG ATC GCC-3′), as described by Ailán-Choke et al. (2020). The thermocycling conditions consisted of an initial denaturation at 94 °C for 5 min, followed by 36 cycles of denaturation at 94 °C for 30 s, annealing at 52.7 °C for 30 s and extension at 72 °C for 1 min, with a final extension at 72 °C for 5 min (Ailán-Choke et al., 2020).

The COI gene was amplified using the primers LCO (forward) (5′-GGT CAA CAA ATC ATA AAG ATA TTG G-3′) and HCO (reverse) (5′-TAA ACT TCA GGG TGA CCA AAA AAT CA-3′), as described by Harnoster et al. (2019). The thermocycling conditions were as follows: initial denaturation at 95 °C for 5 min; 36 cycles of denaturation at 95 °C for 1 min, annealing at 40 °C for 1 min and extension at 72 °C for 1 min 30 s; followed by a final extension at 72 °C for 7 min (Folmer et al., 1994).

PCR reactions for both genes were performed in a final volume of 25 μL, containing 12.5 μL of OneTaq® 2X Master Mix with Standard Buffer, 1.0 μL (10 μM) of each primer, 2.0 μL of genomic DNA (≥20 ng) and 8.5 μL of nuclease-free water. PCR products were quantified, enzymatically purified using ExoProStar™ (GE Healthcare) and submitted for sequencing at ACTGene (Ludwig Biotec, Rio Grande do Sul, Brazil) with the same primers used for PCR amplification.

Contiguous sequences were assembled using Geneious v. 9.1.5 (Biomatters, available from http://www.geneious.com/) and deposited in GenBank. A preliminary BLASTn search was conducted to assess genetic similarity between the newly generated sequences and those of Camallanidae available in the NCBI database (https://www.ncbi.nlm.nih.gov/genbank/).

For phylogenetic analysis, we selected sequences with a minimum length of 500 bp for 18S and 300 bp for COI. We also excluded sequences whose amplified regions did not align with those generated in the present study. In cases where multiple highly similar sequences of the same gene represented a single taxon, often resulting from clones or isolates of a single species within the same study, we retained only one representative sequence.

*Spirocerca lupi* (Rudolphi, 1809) (AY751497) was selected as the outgroup for the 18S dataset, while *Ancyracanthus electrophoris* Virgilio and Pereira, 2023 (OP765585) and *Spiroxys ankarafantsika* Roca and Garcia, 2008 (MW545829) were outgroups for the COI dataset. Table 1 lists the taxa included in the analysis. Although we updated the species names to follow the classification proposed by Silva et al. (2026), the original names recorded in the NCBI database are also provided.

**Table 1.** Camallanid species included in the study, showing updated taxonomic names, corresponding names as listed in the NCBI database, host species, country of record, habitat and gene sequences used in the phylogenetic analyses Table 1 long description.

Species	NCBI name	Host	Country of record	Habitat	18s	COI	Reference
**Subfamily Camallaninae**							
*Camallanus cotti* Fujita, 1927	*Camallanus cotti*	*Awaous guamensis*	New Caledonia	Freshwater	DQ442662	–	Wijová et al. (2006)
		*Lentipes concolor*	–	Freshwater	EF180071	–	Nadler et al. (2000)
		*Raiamas bola*	India	–	–	PV628736	Unpublished
		–	China	Freshwater	–	EU598886, EU598890	Wu et al. (2009)
*Camallanus hypophthalmichthys* Dogel and Akhmerov, 1959	*Camallanus hypophthalmichthys*	*Aristichthys nobilis*	China	Freshwater	JF803915	–	Černotíková et al. (2011)
		–	China	–	–	EU598816	Wu et al. (2009)
*Camallanus kaapstaadi* Southwell and Kirschner, 1937	*Camallanus kaapstaadi*	*Xenopus laevis*	South Africa	–	–	MG948461	Svitin et al. (2018)
*Camallanus lacustris* (Zoega in Müller, 1776)	*Camallanus lacustris*	*Sander lucioperca*	Czech Republic	Freshwater	DQ442663	–	Wijová et al. (2006)
		–	China	Freshwater	OM319832	–	Unpublished
*Camallanus oxycephalus* Ward and Magath, 1916	*Camallanus oxycephalus*	Undetermined fish species	India	MSarine	GU082499, GU082500	–	Unpublished
*Camallanus pallidipectoris* Palumbo et al. 2024	*Camallanus* sp.	*Acanthochelys pallidipectoris*	Argentina	Freshwater	MN936176	–	Palumbo et al. (2024)
*Camallanus xenopodis* Jackson and Tinsley, 1995	*Camallanus xenopodis*	*Xenopus laevis*	South Africa	–	–	MG948462	Svitin et al. (2018)
*Paracamallanus cyathhopharynx* (Baylis, 1923)	*Paracamallanus cyathhopharynx*	*Clarias gaerepinus*	South Africa	Freshwater	OP602982 and OP602986	OP602980 and OP602981	Nofal et al. (2022)
		*Clarias gaerepinus*	South Africa	Freshwater	–	MN523683	Svitin et al. (2019)
*Serpinema cayennensis* Harmoster, Svitin and Du Preez, 2019	*Serpinema cayennensis*	*Rhinoclemmys punctularia*	French Guiana	Freshwater	–	MN104841	Harnoster et al. (2019)
*Zeylanema* sp.	*Zeylanema sp.*	*Mastacembelus erythrotaenia*	Japan	Freshwater	LC813289	LC815080	Saito et al. (2025)
**Subfamily Procamallaninae**							
*Denticamallanus rarus* (Travassos, Artigas and Pereira, 1928)	*Spirocamallanus rarus*	*Calophysus macropterus*	Peru	Freshwater	JF803912	–	Černotíková et al. (2011)
		*Aguarunichthys* cf. *tocantinensis*	Peru	Freshwater	DQ494195	–	Wijová et al. (2006)
*Procamallanus laeviconchus* (Wedl, 1861)	*Procamallanus laevionchus*	*Clarias gariepinus*	Egypt	Freshwater	KP274849	–	Unpublished
		*Synodontis schall*	Sudan	Freshwater	JF803934	–	Černotíková et al. (2011)
		–	Egypt	–	PP581381	–	Unpublished
*Procamallanus pacificus* Moravec, Justine, Wurtz, Taraschewski and Sasal, 2006	*Procamallanus pacificus*	*Anguilla obscura*	New Caledonia	Freshwater	DQ442665	–	Wijová et al. (2006)
*Procamallanus pseudolaeviconchus* Moravec and Van As, 2015	*Procamallanus pseudolaeviconchus*	*Clarias gariepinus*	South Africa	Freshwater	OP602990 and OP602994	OP602967 and OP602969	Nofal et al. (2022)
		*Clarias gariepinus*	South Africa	Freshwater	–	MN523682	Svitin et al. (2019)
*Procamallanus spiculogubernaculus* Agarwal, 1958	*Procamallanus spiculogubernaculus*	*Heteropneustes fossilis*	India	Freshwater	KU292357	KU292358	Chaudhary et al. (2017)
*Procamallanus sigani* Yamaguti, 1935	*Procamallanus sigani*	*Siganus fuscescens*	China	–	HM545908	–	Unpublished
*Procamallanus slomei* Southwell and Kirshner, 1937	*Procamallanus slomei*	*Xenopus laevis*	South Africa	–		MG948463	Svitin et al. (2018)
*Spirocamallanus barlowi* (Santacruz, Barluenga and Pérez-Ponce de León, 2021)	*Procamallanus barlowi*	*Amphilophus citrinellus*	Nicaragua	Freshwater	–	MW487886 and MW487887	Santacruz et al. (2021)
*Spirocamallanus fulvidraconis* (Li, 1935)	*Procamallanus fulvidraconis*	*Pelteobagrus fulvidraconis*	China	Freshwater	DQ076689	–	Wu et al. (2005)
*Spirocamallanus gobiomori* (Moravec, Salgado-Maldonado and Caspeta-Mandujano, 2000	*Procamallanus gobiomori*	*Eleotris* sp.	Mexico	–	–	MT579518	Santacruz et al. (2020)
		*Gobiomorus maculatus*	Mexico	–	–	MT579519	Santacruz et al. (2020)
*Spirocamallanus hilarii* (Vaz and Pereira, 1934)	*Spirocamallanus hilarii*	*Psalidodon endy*	Argentina	Freshwater	MW930855	MW930301	Ailán-Choke and Pereira (2021)
*Spirocamallanus huacraensis* Ramallo, 2008	*Spirocamallanus huacraensis*	*Trichomycterus spegazzinii*	Argentina	Freshwater	MK794615	MK780067	Ailán-Choke et al. (2019)
*Spirocamallanus inopinatus* (Travassos, Artigas and Pereira, 1928)	*Spirocamallanus inopinatus*	*Anostomoides passionis*	Brazil	Freshwater	MT901635	MT898797	Ailán-Choke et al. (2020)
		*Megeleporinus elongatus*	Brazil	Freshwater	MT901634	MT898796	Ailán-Choke et al. (2020)
		*Parupeneus forsskali*	Egypt	Marine	OP563815	–	Unpublished
*Spirocamallanus istiblenni* Noble, 1966	*Spirocamallanus istiblenni*	*Lutjanus fulvus*	–	Marine	EF180076	–	Nadler et al. (2000)
		*Lutjanus kasmira*	Hawaii	Marine	KC505629	KC517404, KC517405	Gaither et al. (2013)
*Spirocamallanus macaensis* (Vicente and Santos, 1972)	*Spirocamallanus macaensis*	*Paralonchurus brasiliensis*	Brazil	Marine	KY436826	–	Sardella et al. (2017)
*Spirocamallanus monotaxis* Olsen, 1952	*Procamallanus monotaxis*	*Lethrinus genivittatus*	New Caledonia		JF803931	–	Černotíková et al. (2011)
*Spirocamallanus neocaballeroi* Caballero-Deloya, 1977	*Procamallanus neocaballeroi*	*Astyanax aeneus*	Mexico	Freshwater	–	MT579517	Santacruz et al. (2020)
		*Astyanax caballeroi*	Mexico	Freshwater	–	MT579514	Santacruz et al. (2020)
*Spirocamallanus xenopodis* (Baylis, 1929)	*Procamallanus xenopodis*	*Xenopus muelleri*	South Africa	–	–	MN523681	Svitin et al. (2019)
*Spirocamallanus philippinensis* Velasquez, 1980	*Spirocamallanus philippinensis*	*Siganus guttatus*	Phillippines	–	JF934736	–	Laetsch et al. (2012)
*Spirocamallanus pintoi* Kohn and Fernandes, 1988	*Procamallanus pintoi*	*Corydoras atropersonatus*	Peru	Freshwater	DQ442666,	–	Wijová et al. (2006)
		*Corydoras micracanthus*	Argentina	Freshwater	MW930856	MW930302	Ailán-Choke and Pereira (2021)
*Spirocamallanus rebecae* Andrade-Salas, Pineda-López and Garcia-Magaña, 1994	*Procamallanus rebecae*	*Cichlasoma meeki*	Mexico	Freshwater	DQ442667	–	Wijová et al. (2006)
		*Cribroheros robertsoni*	Mexico	Freshwater	–	MW487880	Santacruz et al. (2021)
		Cichlid	Mexico	Freshwater	–	MW487881	Santacruz et al. (2021)
**Outgroups**							
*Ancyracanthus electrophoris* Virgilio and Pereira, 2023	*Ancyracanthus electrophoris*	*Electrophorus varii*	Brazil	Freshwater	–	OP765585	Virgilio et al. (2023)
*Spirocerca lupi* (Rudolphi, 1809)	*Spirocerca lupi*	*Urocyon littoralis*	USA	–	AY751497	–	Unpublished^a^
*Spiroxys ankarafantsika* Roca and Garcia, 2008	*Spiroxys ankarafantsika*	*Pelusios sinuatus, P. subniger* and *P. castanoides*	Mozambique and South Africa	Freshwater	–	MW545829	Nel et al. (2021)

*Notes*: ^a^Used as in Ailán-Choke and Pereira (2021).

Our sequences were aligned using MUSCLE (Edgar, 2004) with default parameters implemented in Geneious v. 7.1.3. After trimming the terminal regions of the alignments, we assessed the presence of stop codons using the second translation frame under the invertebrate mitochondrial genetic code in Geneious (Kearse et al., 2012).

Substitution saturation in the aligned dataset was assessed using the ISS index in DAMBE 7 (Xia, 2018). Genetic divergence estimates, including pairwise base substitution rates per site, were calculated in MEGA11 (Kimura, 1980; Tamura et al., 2021), with standard errors estimated using a bootstrap procedure with 1000 replicates. Model selection based on the Akaike Information Criterion identified GTR + I + G as the best-fitting nucleotide substitution model, which was subsequently applied in phylogenetic analyses.

We performed phylogenetic reconstructions using maximum likelihood (ML) in RAxML v. 8.2.12 and Bayesian inference (BI) in MrBayes v. 3.2.7a (Guindon and Gascuel, 2003; Ronquist et al., 2003). Both analyses were performed in the CIPRES Science Gateway (Miller et al., 2010). ML analyses included 1000 bootstrap replicates, and we considered only nodes with bootstrap support values greater than 70% as well-supported clades.

BI employed the following settings: *lset* nst = 6, rates = invgamma, ngammacat = 4, nucmodel = 4by4, code = universal and *prset* statefreqpr = Dirichlet (1,1,1,1). Markov Chain Monte Carlo analyses were run for 10 000 000 generations, sampling one tree every 1500 generations. The initial 25 000 generations were discarded as burn-in. A majority-rule consensus tree was generated from the remaining topologies, using the commands sumt relburnin = yes and sump relburnin = yes. Only nodes with Bayesian posterior probabilities greater than 90% were considered well supported. Trees were visualized and edited in FigTree v. 1.3.3 (Rambaut, 2009).

## Results

### Taxonomic summary

Family Camallanidae Railliet and Henry, 1915

Subfamily Camallaninae Yeh, 1960

Genus *Serpinema* Yeh, 1960

**Figure 1.**
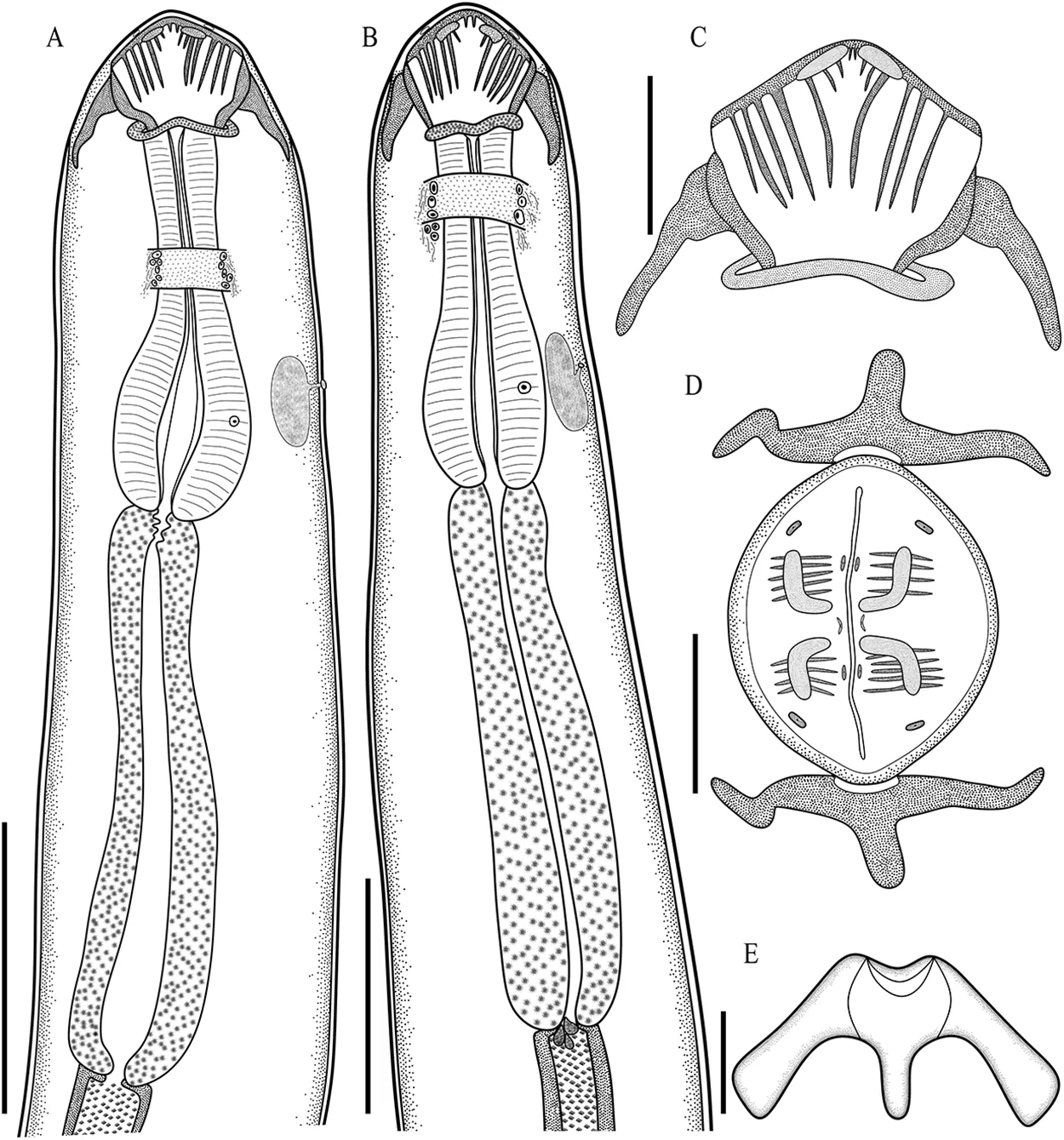
Line drawings of the anterior region of *Serpinema yehi* n. sp. Silva, Tavares-Costa and Melo, parasite of *Rhinoclemmys punctularia* from the Brazilian Amazon. (A) Anterior region of male, ventral view. (B) Anterior region of female, ventral view. (C) Valve of buccal capsule, ventral view. (D) Cephalic extremity, apical view. (E) Trident, ventral view. Scale bars: (A and B) 350 µm; (C and D) 100 µm; (E) 50 µm.

**Figure 2.**
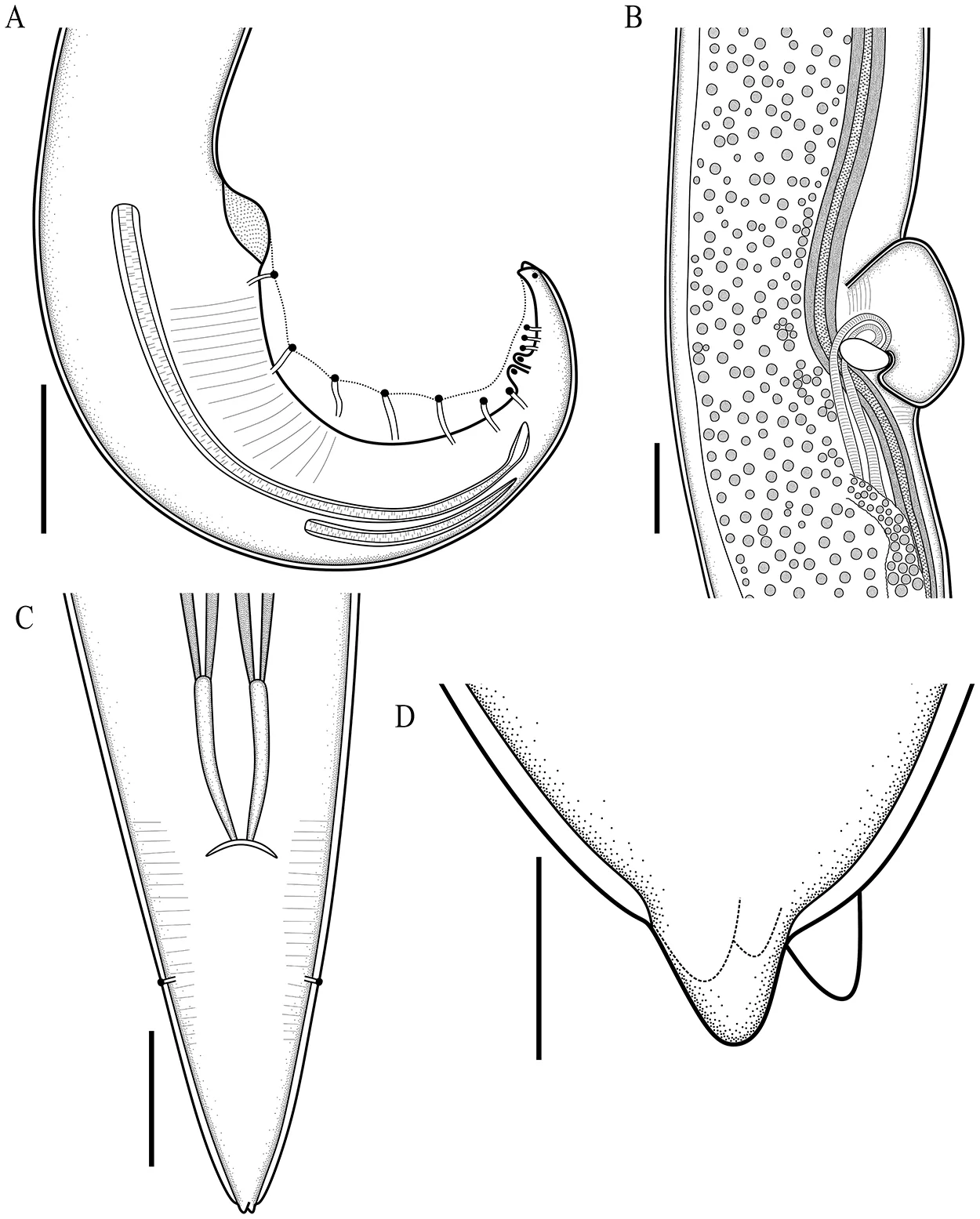
Line drawings of the posterior region of *Serpinema yehi* n. sp. Silva, Tavares-Costa, and Melo, parasite of *Rhinoclemmys punctularia* from the Brazilian Amazon. (A) Posterior region of male, showing caudal papillae and spicules, lateral view. (B) Vulva, lateral view. (C) Posterior region of female, ventral view. (D) Posterior extremity of female tail, showing the four mucrons, ventrolateral view. Scale bars: (A and B) 200 µm; (C) 100 µm; (D) 15 µm.

**Figure 3.**
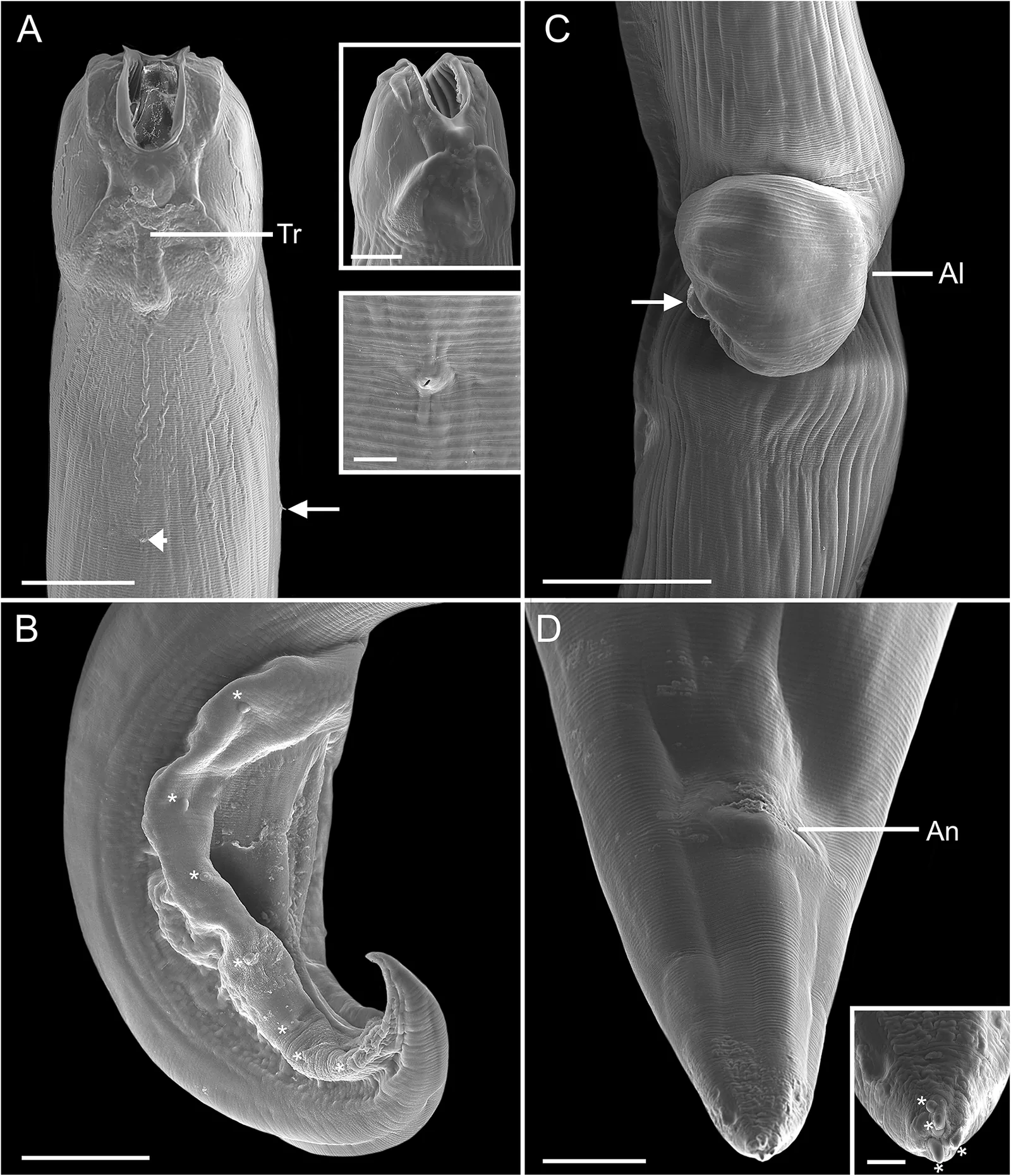
Scanning electron microscopy of *Serpinema yehi* n. sp. Silva, Tavares-Costa, and Melo, parasite of *Rhinoclemmys punctularia* from the Brazilian Amazon. (A) Anterior region, ventral view, showing deirid (arrow) and excretory pore (arrowhead). Inserts: Lateroventral view of the anterior region and detail of excretory pore. (B) Posterior region of male, lateroventral view, showing precloacal papillae (asterisk). (C) Vulva, ventral view, showing the anterior lip and the small lateral projection (arrow). (D) Posterior region of female, subventral view. Insert: detail of tip of female showing four mucrons (asterisk). Abbreviations: Al, anterior lip; An, anus; Tr, trident. Scale bars: (A) 100 µm, Insert, 50 µm (anterior region), 10 µm (excretory pore); (B) 100 µm; (C) 200 µm; (D) 50 µm, Insert, 10 µm.

***Serpinema yehi* n. sp. Silva, Tavares-Costa, and Melo (Figs. 1**, **2**, **3**).

Type host: *Rhinoclemmys punctularia* (Daudin, 1801).

Type locality: Abaetetuba (01°43’46” S 48°52’27” W), Pará, Brazil.

Site of infection: Stomach, small intestine and large intestine.

Prevalence: 100% (4 hosts infected out of 4 examined).

Mean abundance and mean intensity: both 35.75 (143 specimens recovered, including 71 males and 72 females).

Specimens deposited: Holotype (MPEG.NEM 000423), allotype (MPEG.NEM 000426), 9 male paratypes (MPEG.NEM 000424), 9 female paratypes (MPEG.NEM 000425) and voucher hologenophore (MPEG.NEM 000427) deposited in the Other Invertebrate Collection of Museu Paraense Emílio Goeldi, Pará, Brazil.

ZooBank registration: To comply with the regulations set out in article 8.5 of the amended 2012 version of the International Code of Zoological Nomenclature (ICZN, International Commission on Zoological Nomenclature, 2012), details of the new species have been submitted to ZooBank. The Life Science Identifier for *Serpinema yehi* n. sp. is urn:lsid:zoobank.org:act:C92AF89C-C3E2-4D72-A349-A3F94DF44316.

Representative DNA sequences: GenBank [PZ113237] (18S); [PZ113265] (COI).

Etymology: The specific epithet honors Yeh Liang-Sheng in recognition of his significant contributions to parasitology, particularly to the systematics of the family Camallanidae.

### Description

General: Large nematodes. Reddish in life. Cuticle bearing fine transverse striations along entire body length. Anterior extremity truncated. Lips absent. Oral opening as transverse slit; one pair of lateral amphids; two pairs of small cephalic papillae close to oral opening; two pairs of cephalic papillae more distant from oral opening. Two apical pairs of curved sclerotized plates. Buccal capsule composed of two laterally positioned, internally concave valves (Figure 1D). Valves internally with longitudinal complete ridges (extending from anterior to posterior margin of buccal capsule) and incomplete ridges (extending from anterior margin to before midlength of buccal capsule) (Figure 1C). Two tridents, dorsal and ventral (Figure 1D), slightly rounded basal portion, 3 divergent branches; median branch shorter, less robust; lateral branches similar (Figures 1E, 3A). Chitinous basal ring surrounding junction between buccal capsule and oesophagus. Oesophagus elongate, divided; anterior muscular region short; posterior glandular region long. Nerve ring at midlength of muscular oesophagus. Excretory pore at level of posterior half of muscular oesophagus. Deirids posterior to excretory pore (Figure 3A).

Males (based on holotype and 9 paratypes): Total length 10.92 (10.70, 9.72–11.73) mm. Body width at midbody 307 (332, 267–387). Sclerotized plates 9 (14, 9–18) × 40 (42, 34–47). Buccal capsule 122 (124, 118–129) × 153 (152, 143–162). Buccal valves with 9 (10, 8–11) complete ridges, 6 (4, 3–7) incomplete ridges. Median trident branch 69 (81, 68–100) long; lateral trident branches 98 (101, 94–113) long. Basal ring 16 (17, 13–21) × 103 (105, 99–116). Oesophagus 1.283 (1.246, 1.157–1.328) mm long. Muscular oesophagus 512 (480, 435–525) × 152 (168, 144–181). Glandular oesophagus 771 (767, 717–813) × 168 (195, 160–237). Nerve ring, excretory pore, and deirids 245 (242, 221–284), 464 (417, 309–485), and 507 (501, 453–555), respectively, from anterior end (Figure 1A). Tail ventrally curved, ending with two tips, 160 (192, 157–227) long; strong transverse musculature. Caudal alae well developed, beginning at level of globose, vesicle-like swelling, ending near tail tip. Twelve pairs of caudal papillae. Seven pedunculate precloacal papillae, all lateral (Figure 3B). Two adcloacal, one pair on anterior cloacal lip, one on posterior cloacal lip. Three pedunculate postcloacal, all lateral, clustered near cloaca. Phasmids present, near posterior end. Gubernaculum absent. Spicules unequal in length and shape; right spicule 869 (800–891) long, posterior end spatulate or leaf-like in shape; left spicule 389 (373–453) long, with tapered posterior end (Figure 2A).

Females (based on allotype and 9 paratypes): Total length 17.16 (16.65, 15.24–18.00) mm. Body width at midbody 440 (455, 413–507). Sclerotized plates 16 (17, 14–20) × 52 (52, 48–57). Buccal capsule 151 (151, 132–160) × 197 (191, 182–199). Buccal valves with 10 (10, 9–11) complete ridges, 4 (4, 1–6) incomplete ridges. Median trident branch 110 (102, 82–127) long; lateral trident branches 121 (126, 110–141) long. Basal ring 19 (20, 17–22) × 129 (130, 119–147). Oesophagus 1.435 (1.391, 0.94–1.53) mm long. Muscular oesophagus 517 (560, 517–592) × 200 (197, 184–213). Glandular oesophagus 864 (875, 803–939) × 245 (225, 181–253). Nerve ring, excretory pore and deirids 280 (286, 261–304), 552 (472, 371–563) and 563 (567, 509–677), respectively, from anterior end (Figure 1B). Vulva near midbody; located 8.16 (8.37, 7.53–8.77) mm from posterior extremity; anterior lip well developed, bearing small lateral projection near vulvar opening (Figures 2B, 3C); posterior lip indistinct. Ovoviviparous. Tail 301 (337, 237–544) long (Figure 2C); ending in 4 tips or mucrons, 2 ventrolateral, 1 dorsal and 1 small dorsolateral (Figures 2D, 3D). Phasmidial pores 184 (181, 157–213) from posterior end (Figure 2C).

### Molecular analyses results

We obtained a 583 bp sequence of the mitochondrial COI gene of *Se. yehi* n. sp., whereas the ribosomal 18S gene yielded a sequence of 833 bp. BLASTn similarity searches revealed no identical matches (100% identity) within Camallanidae sequences currently available in the NCBI database. After aligning our sequences with those from GenBank, we generated three distinct matrices: a 505 bp matrix for the 18S gene and a 340 bp matrix for the COI gene, both of which included representative taxa from the family Camallanidae. Additionally, we constructed a 427 bp COI gene matrix comprising only species from the subfamily Camallaninae.

**Figure 4.**
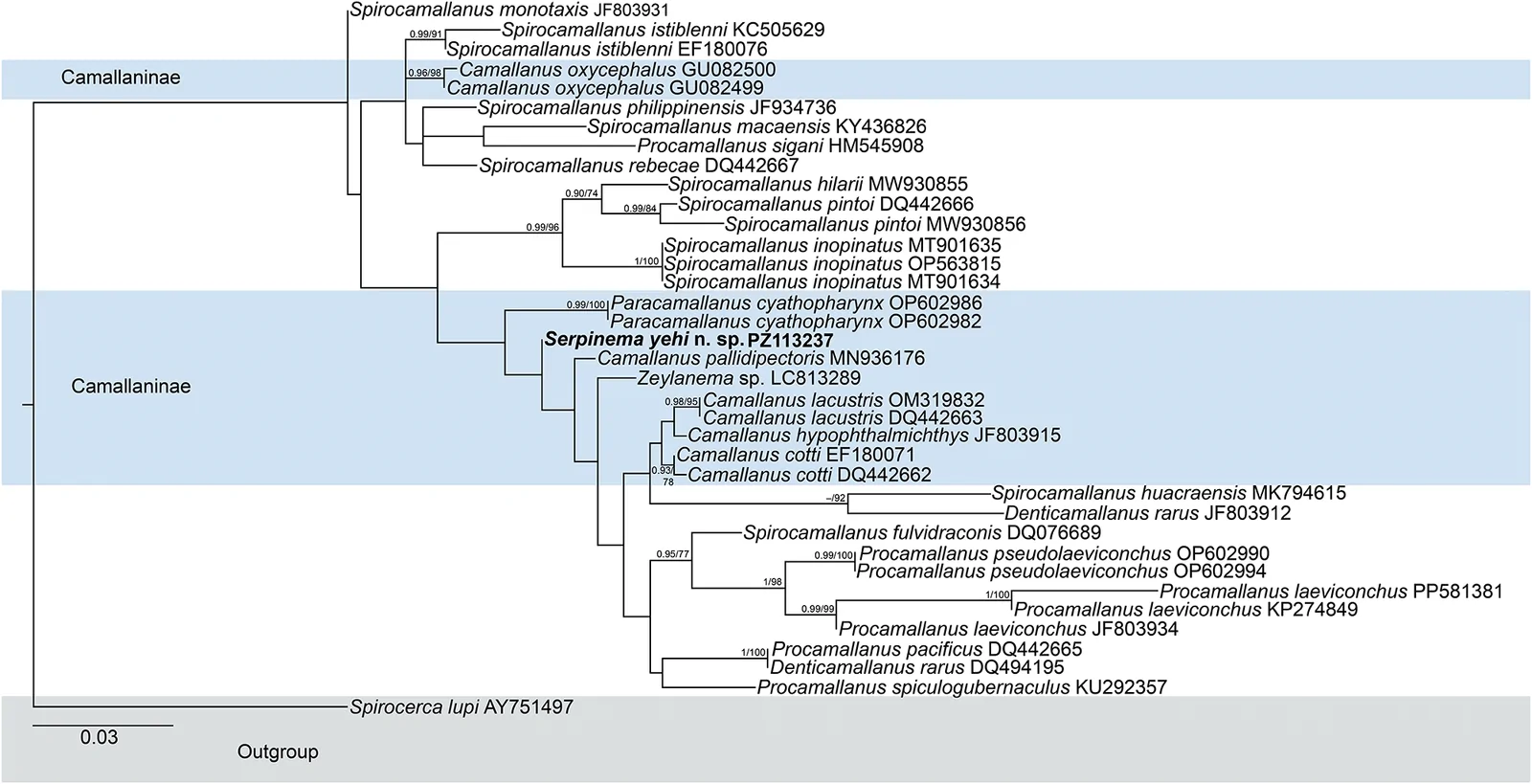
Maximum likelihood phylogenetic tree of Camallanidae species based on the 18S gene, using *Spirocerca lupi* as outgroup. The position of *Serpinema yehi* n. sp. is indicated in bold italics. GenBank accession numbers are provided after each taxon. Node support values are shown above or below branches; posterior probabilities <0.90 and bootstrap values <70 are either not shown or indicated by a dash. The branch-length scale bar represents the number of substitutions per site. Figure 4 long description.

We did not find any 18S gene sequences of *Serpinema* species available in the NCBI database. Thus, *Se. yehi* n. sp. appeared more closely related to *Camallanus pallidipectoris*, a parasite of the freshwater turtle *Acanthochelys pallidipectoris* from Argentina, and to *Zeylanema* sp., a parasite of the freshwater fish *Mastacembelus erythrotaenia* from Japan (Figure 4). The new species exhibited genetic divergence of up to 10% compared with Camallanidae sequences available in GenBank. The lowest divergence (1%) was observed between *Se. yehi* n. sp. and sequences MN936176 (*C. pallidipectoris*) and LC813289 (*Zeylanema* sp.), whereas the highest (10%) occurred relative to sequence PP581381 (*Procamallanus laeviconchus*). Interestingly, divergence between *Se. yehi* n. sp. and *Pr. laeviconchus* was greater than that observed in relation to the outgroup *Spirocerca lupi* (AY751497) (7%). Genetic divergence values among sequences are presented in Supplementary Material 1.

Our phylogenetic analysis of the 18S gene recovered four well-supported subclades comprising more than one species. The first subclade includes *Spirocamallanus hilarii*, from *Psalidodon endyi* (freshwater) from Argentina, *Sp. pintoi*, recorded from *Corydoras atropersonatus* (Peru) and *Corydoras micracanthus* (Argentina) (both freshwater), together with *Sp. inopinatus*, parasitizing *Anostomoides passionis* and *Megaleporinus elongatus* (freshwater) from Brazil and *Parupeneus forssalis* from Egypt (marine).

The second subclade includes *Denticamallanus rarus*, a parasite of *Trichomycterus spegazzinni* (freshwater) from Argentina and *Sp. huacraensis*, a parasite of *Calophysus macropterus* (freshwater) from Peru. The third subclade comprises *Sp. fulvidraconis*, a parasite of *Pelteobagrus fulvidraconis* (freshwater) from China; *Pr. pseudolaeviconchus*, from *Clarias gariepinus* from South Africa, and *Pr. laeviconchus*, recorded from *Clarias gariepinus* in Egypt and from *Synodontis schall* in Sudan (all freshwater). Finally, the fourth subclade groups *D. rarus*, parasitizing *Aguarunichthys* cf. *tocantinensis* (freshwater) from Peru, and *Pr. pacificus*, from *Anguilla obscura* (freshwater) from New Caledonia (Figure 4).

Nine well-supported subclades composed of a single species were recovered: *Sp. istblenni*, a parasite of *Lutjanus kasmira* from Hawaii and *Lutjanus fulvus* (both marine); *C. oxycephalus*, parasitizing marine fishes from India; *Sp. pintoi*, parasitizing *Corydoras atropersonatus* from Peru and *Corydoras micracanthus* from Argentina (both freshwater); *Sp. inopinatus*, parasitizing *Anostomoides passionis* and *Megaleporinus elongatus* (freshwater) from Brazil and *Parupeneus forsskali* from Egypt (marine); *Paracamallanus cyathopharynx*, from *Clarias gariepinus* in South Africa (freshwater); *C. lacustris*, parasitizing *Sander lucioperca* (freshwater) and an unidentified freshwater fish from China; *C. cotti*, parasitizing *Lentipes concolor* (freshwater); *Pr. pseudolaeviconchus*, also from *Clarias gariepinus* in South Africa (freshwater); *Pr. laeviconchus*, recorded from *Clarias gariepinus* in South Africa and from an unidentified freshwater fish in Egypt. The subclades corresponding to *Sp. inopinatus* and *Sp. pintoi* are nested within a larger clade composed of *Sp. inopinatus, Sp. hilarii* and *Sp. pintoi*. Likewise, the subclades of *Pr. pseudolaeviconchus* and *Pr. laeviconchus* are included within the clade formed by *Sp. fulvidraconis, Pr. pseudolaeviconchus* and *Pr. laeviconchus* (Figure 4).

**Figure 5.**
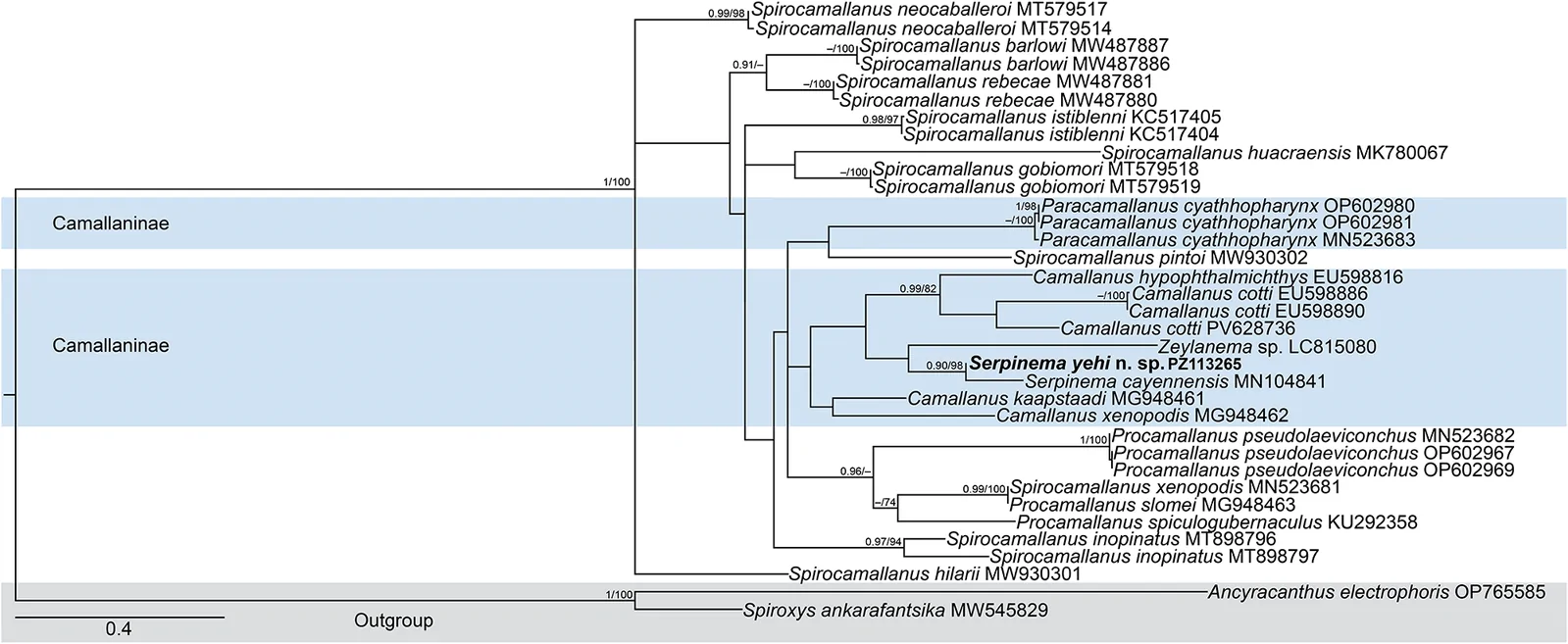
Maximum likelihood phylogenetic tree of Camallanidae species based on the COI gene, using *Ancyracanthus electrophoris* and *Spiroxys ankarafantsika* as outgroups. The position of *Serpinema yehi* n. sp. is indicated in bold italics. GenBank accession numbers are provided after each taxon. Node support values are shown above or below branches; posterior probabilities <0.90 and bootstrap values <70 are either not shown or indicated by a dash. The branch-length scale bar represents the number of substitutions per site. Figure 5 long description.

In the phylogenetic analysis of the COI gene, *Se. yehi* n. sp. clustered with *Se. cayennensis*, a parasite of *Rhinoclemmys punctularia* from French Guiana. Together, these taxa formed a well-supported clade representing the genus *Serpinema* (Figure 5). The new species exhibited high genetic divergence compared with other Camallanidae taxa available in GenBank. The lowest divergence (6%) was observed with the only other congener, *Se. cayennensis* (MN104841), whereas the highest divergence (21%) occurred between *Se. yehi* n. sp. and *Pr. pseudolaeviconchus* (OP602967 and OP602969). In comparison with the outgroups, divergence values were 38% and 43% relative to *Spiroxys ankarafantsika* (MW54582) and *Ancyracanthus electrophoris* (OP765585), respectively. Genetic divergence values among sequences are presented in Supplementary Material 2.

Based on the COI matrix for Camallanidae, we obtained four well-supported subclades comprising multiple species. The first subclade includes *Sp. barlowi*, a parasite of *Amphilophus citrinellus* from Nicaragua, and *Sp. rebecae*, which parasitizes cichlids, including *Cibroheros robertsoni*, in Mexico (freshwater). The second subclade comprises *C. cotti* from China, specimens infecting *Raiamas bola* in India, and *C. hypophthalmichtys*, also from China. The third subclade corresponds to the clade formed by *Serpinema yehi* n. sp and *Se. cayennensis*. The fourth subclade grouped *Sp. xenopodis*, parasitic in *Xenopus muelleri* and *Pr. slomei*, from *Xenopus laevis*, both recorded in South Africa. This subclade is nested within a larger, well-supported clade comprising *Sp. xenopodis, Pr. slomei, Pr. spiculogubernaculus* (a parasite of *Heteropneustes fossilis* from India) and *Pr. pseudolaeviconchus* (from *Clarias gariepinus* in South Africa) (Figure 5).

We also obtained nine single-species subclades with strong support. These included: *Sp. neocaballeroi*, parasitizing *Astyanax aeneus* and *Astyanax caballeroi* in Mexico; *Sp. barlowi*, from *Amphilophus citrinellus* in Nicaragua; *Sp. rebecae*, infecting cichlids, including *Cibroheros robertsoni*, in Mexico; *Sp. istblenni*, a parasite of *Lutjanus kasmira* from Hawaii; *Sp. gobiomori*, recorded from *Eleotris* sp. and *Gobiomorus maculatus* in Mexico; *Pa. cyathopharynx*, from *Clarias gariepinus* in South Africa; *C. cotti*, from China; *Pr. pseudolaeviconchus*, also parasitizing *Clarias gariepinus* in South Africa; and *Sp. inopinatus*, infecting *Megaleporinus elongatus* and *Anostomoides passionis* in Brazil (Figure 5).

**Figure 6.**
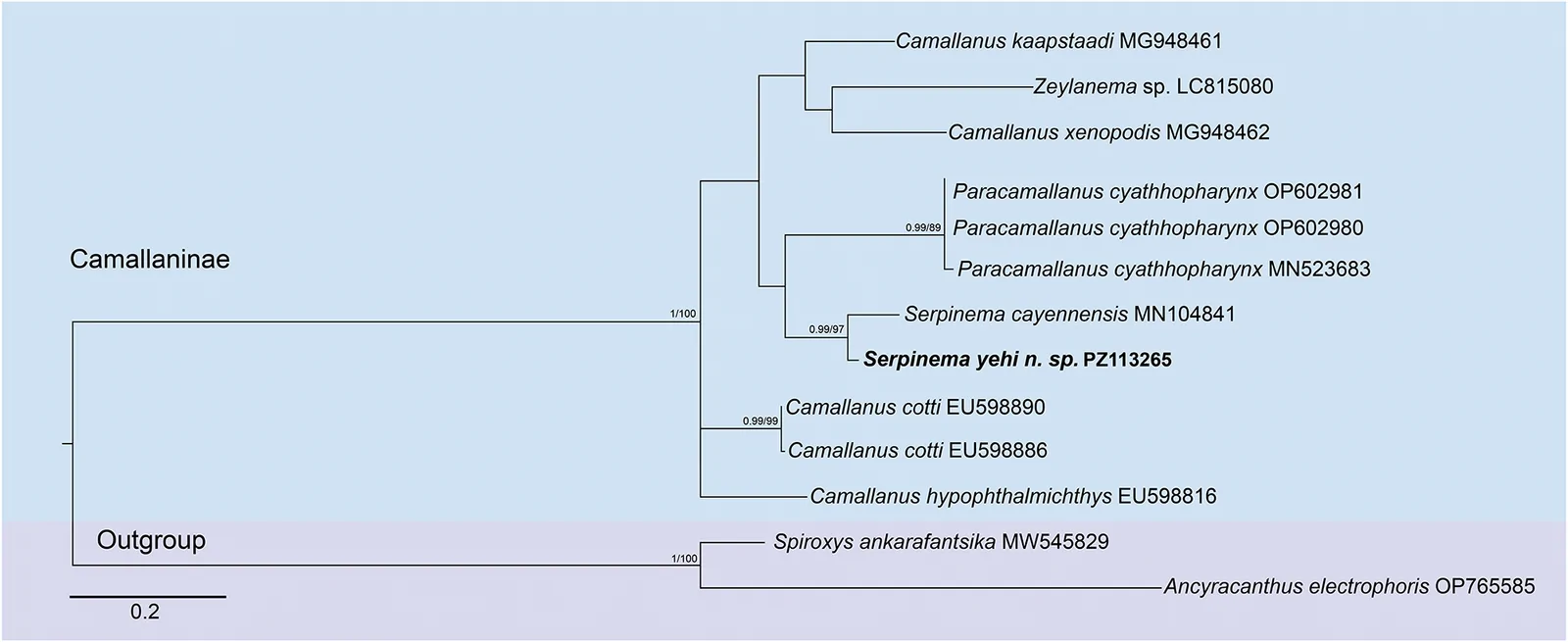
Maximum likelihood phylogenetic tree of species of the subfamily Camallaninae based on the COI gene, using *Ancyracanthus electrophoris* and *Spiroxys ankarafantsika* as outgroups. The position of *Serpinema yehi* n. sp. is indicated in bold italics. GenBank accession numbers are provided after each taxon. Node support values are shown above or below branches; posterior probabilities <0.90 and bootstrap values <70 are either not shown or indicated by a dash. The branch-length scale bar represents the number of substitutions per site. Figure 6 long description.

To more accurately evaluate relationships within the subfamily Camallaninae, we conducted an analysis using representative COI sequences from this group. Three well-supported subclades were recovered: the *Pa. cyathopharynx* subclade, the *C. cotti* subclade and the *Serpinema* clade, comprising *Se. yehi* n. sp. and *Se. cayennensis* (Figure 6). In this dataset, *Se. yehi* n. sp. exhibited high genetic divergence relative to the remaining sequences, ranging from 7% compared with *Se. cayennensis* (MN104841) to 18% compared with *C. xenopodis* (MG948462) and *Pa. cyathopharynx* (MN523683, OP602980 and OP602981). Divergence from the outgroups reached 32% and 38% relative to *Spiroxys ankarafantsika* (MW545829) and *Ancyracanthus electrophoris* (OP765585), respectively. Genetic divergence values among all sequences are presented in Supplementary Material 3.

### Remarks

The specimens were assigned to *Serpinema* based on the presence of a buccal capsule composed of two lateral valves with internal longitudinal ridges arranged into two groups, dorsal and ventral, separated by a distinct gap. These features correspond to the diagnostic characters of the genus as defined by Yeh (1960) and Silva et al. (2026).

Following Silva et al. (2023), *Serpinema* spp. can be divided into two morphogroups based on the number of spicules. Morphogroup I includes species with a single spicule (*Se. lissemysus, Se. magathi, Se. monospiculatum* and *Se. pelliculatum*), whereas morphogroup II comprises species with two unequal spicules (*Se. amazonicum, Se. cayennensis, Se. emydidium, Se. intermedius, Se. kachugae, Se. microcephalus, Se. trispinosum* and *Se. octorugatum*). Thus, *Se. yehi* n. sp. belongs to morphogroup II. Accordingly, the new species was compared with other congeners assigned to this morphogroup, regardless of geographic distribution.

Based on the original description of *Se*. *amazonicum* by Ribeiro (1941) of specimens collected from *Podocnemis expansa* (Schweigger, 1812) (Podocnemididae) in Brazil, *Se*. *yehi* n. sp differs by having fewer buccal capsule ridges (11–18 in males, 10–17 in females in *Se. yehi* n. sp. vs. 17–20 in both sexes) and a longer tail in both sexes (157–227 and 237–544 vs. 130 and 210 for males and females, respectively). Males of *Se. yehi* n. sp. further differ by having longer spicules (800–891 and 373–453 vs. 570 and 240 for right and left spicules, respectively), and a different number and arrangement of pairs of caudal papillae (7:2:3 vs. 7:0:2). Females can additionally be distinguished by the morphology of vulvar lips; in the new species, the anterior lip is well developed, and the posterior vulvar lip is indistinct (vs. either a prominent anterior or posterior lip in *Se. amazonicum*), as well as by the presence of 4 mucrons at the tail tip (vs. 3 in *Se. amazonicum*).

*Serpinema yehi* n. sp. can be distinguished from *Se. cayennensis*, a parasite of *Rhinoclemmys punctularia* (same type host for both species) (Geoemydidae) from French Guiana (Harnoster et al., 2019), by the greater total length (800–891 vs. 360–806) and distinct morphology of the right spicule (leaf-like posterior end vs. bearing a short process near the distal extremity), as well as by the number of caudal papillae (7:2:3 vs. 6:2:5). Females also differ by the number of the mucrons at tail tip (4 vs. 3).

Based on the description of *Se. emydidium* by Mascarenhas and Müller (2017) from *Trachemys dorbigni* (Duméril and Bibron, 1835) (Emydidae) in Brazil, the new species differs by the number of caudal papillae (7:2:3 vs. 7:2:4), right spicule morphology (flattened extremity vs. simple distal extremity) and the number of mucrons at the female tail tip (4 vs. 3). Although specimens of *Se. emydidium* are generally smaller, they may reach body lengths comparable to those of the new species (9.72–11.73 and 15.24–18.00 mm in *Se. yehi* n. sp. vs. 6.55–11.4 and 11.28–18.71 mm for males and females, respectively) and exhibit a slightly longer right spicule (800–950 vs. 800–891 in the new species).

*Serpinema yehi* n. sp. can be easily distinguished from *Se. intermedius*, a parasite of *Mauremys reevesii* (Gray, 1831) (Geoemydidae) from China (Ivashkin et al., 1977), by the number and arrangement of caudal papillae (7:2:3 vs. 7:2:5) and morphology of the right spicule. In *Se. intermedius*, the right spicule ends in a bifid tip, whereas in the new species terminate in leaf-like extremity, with one tip. Furthermore, females differ by the presence of phasmids in *Se. yehi* n. sp. (vs. not observed) and by vulva morphology, with a well-developed anterior lip and indistinct posterior lip in the new species, whereas both lips are indistinct in *Se. intermedius*.

Based on the original description of specimens collected from *Pangshura smithii* (Gray, 1863) (Geoemydidae) in India (Baylis and Daubney, 1922), *Se*. *kachugae* can be distinguished from *Se. yehi* n. sp. by the smaller number of buccal capsule ridges in both sexes (8–10 in both sexes in *Se. kachugae* vs. 11–18 in males and 10–17 in females), a greater number of caudal papillae pairs (7:2:6 vs. 7:2:3 in the new species), and a longer right spicule (970 vs. 800–891 in *Se. yehi* n. sp.). Females of the new species also differ in their smaller body length (15.24–18.00 mm vs. 20.8–22.0 mm in *Se. kachugae*), vulva lip morphology, characterized by a well-developed anterior lip and indistinct posterior lip (vs. prominent vulvar lips, with the anterior considerably larger and overlapping the posterior) and by the presence of 4 mucrons at the tail tip (vs. 2).

Compared with *Se. microcephalus*from the original description by Dujardin (1845) of specimens collected from *Emys orbicularis* (Linnaeus, 1758) (Emydidae) in France, *Serpinema yehi* n. sp. differs by having a higher number of buccal capsule ridges (11–18 in males and 10–17 in females vs. 10 in both sexes in *Se. microcephalus*) and a different number and arrangement of caudal papillae pairs (7:2:3 in *Se. yehi* n. sp. vs. 7:0:5). In addition, the new species exhibits larger females (15.24–18.00 vs. 11.68–12.73 mm) and a longer tail in both sexes (157–227 and 237–544 in the new species vs. 62 and 172 for males and females, respectively).

Based on the redescription by Sharma et al. (2002) of specimens collected from *Cuora amboinensis* (Daudin, 1801) (Geoemydidae) in Malaysia, *Se*. *octorugatum* differs from the new species by the arrangement of caudal papillae (7:2:3 in *Se. yehi* n. sp. vs. 7:2:5) and right spicule morphology. In *Se. octorugatum*, the right spicule is U-shaped in cross-section, ventrally flexed, bearing a deep ventral groove and a complex lamella-like formation near the distal end, followed by abrupt flattening. In contrast, that of *Se. yehi* n. sp. is simple, with a leaf-like posterior end. Females differ by the number of mucrons at the tail tip (4 vs. 3 in *Se. octorugatum*).

*Serpinema yehi* n. sp. further differs from *Se. trispinosum*, described by Moravec and Vargas-Vázquez (1998) based on specimens collected from *Trachemys scripta* (Thunberg, 1792) (Emydidae) in Mexico, by the number and arrangement of caudal papillae (7:2:3 in the new species vs. 7:0:6) and by its larger body size in both males (9.72–11.73 mm vs. 4.43–7.53 mm in *Se. trispinosum*) and females (15.24–18.00 mm vs. 7.47–10.35 mm). Females also differ in the number of mucrons at the tail tip (4 in the new species vs. 3 in *Se. trispinosum*).

Although the new species belongs to morphogroup II (with two spicules), we also compared it with three species assigned to morphogroup I (*Se. magathi, Se. monospiculatum* and *Se. pelliculatum*). We included these species in our analysis because they also parasitize freshwater turtles in Brazil, mainly *Kinosternon scorpioides* (Linnaeus, 1766) (Izidro de Brito and Lacerda, 2025).

Thus, *Se. yehi* n. sp. can be distinguished from *Se. magathi*, reported from *Kinosternon scorpioides* (Kinosternidae) in the state of Pernambuco, northeastern Brazil (Freitas and Dobbin Jr, 1971), by spicule morphology (leaf-like posterior end in *Se. yehi* n. sp. vs. bifid distal tip bearing unequal branches), number and arrangement of caudal papillae (7:2:3 in new species vs. 7:0:7), vulvar lip morphology (well-developed anterior lip and indistinct posterior lip in new species vs. 2 prominent lips) and number of mucrons on female tail (4 vs. 3 in *Se. magathi*).

The new species also differs from *Se. monospiculatum*, described from specimens obtainded from *Mesoclemmys tuberculata* (Luederwaldt, 1926) (Chelidae) in the state of Pernambuco, northeastern Brazil (Freitas and Dobbin Jr, 1971), by spicule morphology (leaf-like posterior extremity in *Se. yehi* n. sp. vs. a filiform tip after curvature), number and arrangement of caudal papillae (7:2:3 in new species vs. 6:1:3), vulvar lip morphology (well-developed anterior lip and indistinct posterior lip vs. two non-prominent lips in *Se. monospiculatum*) and number of mucrons on female tail (4 vs. 3).

*Serpinema yehi* n. sp. can be distinguished from *Se. pelliculatum*, described from *K. scorpioides* (Kinosternidae) in Pará state, northern Brazil, Amazon region (Silva et al., 2023), by spicule morphology (leaf-like posterior end, without ornamentations vs. covered by a sheath in *Se. pelliculatum*), number of caudal papillae (7:2:3 in *Se. yehi* n. sp. vs. 7:2:6), vulvar lip morphology (well-developed anterior lip and indistinct posterior lip vs. both lips prominent, with the posterior lip more developed than the anterior in *Se. pelliculatum*) and number of mucrons on female tail (4 in the new species vs. 3).

Thus, *Se. yehi* n. sp. can be distinguished from its congeners by the number of buccal capsule ridges, the number and arrangement of caudal papillae, the morphology and length of the spicules, the morphology of the vulvar lip and the number of mucrons on the female tail. Additional morphometric differences between *Se. yehi* n. sp. and its congeners are provided in Table 2.

**Table 2.** Host species, country of record and main morphological and morphometric characteristics of the 13 valid species of Serpinema, organized by morphogroup Table 2 long description.

Morphogroup I									
	*Serpinema lissemysus* (Gupta and Singh, 1959)		*Serpinema magathi* (Sprehn, 1932)		*Serpinema pelliculatum* Silva, Jesus and Melo, 2023			*Serpinema monospiculatum* Freitas and Dobbin Jr., 1962	
Reference	Ivashkin et al. (1977)		Freitas and Dobbin Jr (1971)		Silva et al. (2023)			Freitas and Dobbin Jr (1971)	
Host	*Lissemys punctata*		*Kinosternon scorpioides*		*Kinosternon scorpioides*			*Mesoclemmys tuberculata*	
Country	India		Brazil		Brazil			Brazil	
Number of ridges	♂ 14−16		♂ 9−11		♂ 9−11 ♂			♂ 10−12	
	♀ 14−16		♀ 9−11		♀ 9−11			♀ 10−12	
Pairs of caudal papillae (pre:ad:post)	6:2:10		7:0:7		7:2:6			6:1:3	
Total length (mm)	♂ 4.75−5.00		♂ 8.34−8.93		♂ 6.07−8.20			♂ 9.55−10.92	
	♀ 10.5		♀ 12.58		♀ 8.05−9.91			♀ 17.08−18.69	
Tail	♂ 130−170		♂ 90−130		♂ 83−131			♂ 90−130	
	♀ –		♀ 220		♀ 140−197			♀ 200−230	
Spicule	–		540−590		416−566			430	
Vulva (mm)	–		7.13		3.71−4.64			6.21−8.87	
Vulva lips morphology	Anterior lip larger than the posterior		Prominent lips		Prominent lips; posterior one more developed			Non-prominent lips	
Phasmids	–♂		–♂		–♂			–♂	
	–♀		–♀		–♀			–♀	
Mucrons on female tail	–		3		3			3	

Morphogroup II									
	*Serpinema yehi* Silva, Tavares-Costa and Melo n. sp.	*Serpinema amazonicum* (Ribeiro, 1941)	*Serpinema cayennensis* Harnoster, Svitin and Du Preez, 2019	*Serpinema emydidium* (Mascarenhas and Müller, 2017)	*Serpinema intermedius* (Hsu and Hoeppli, 1931)	*Serpinema kachugae* (Baylis and Daubney, 1922)	*Serpinema microcephalus* (Dujardin, 1845)	*Serpinema octorugatum* (Baylis, 1933)	*Serpinema trispinosum* (Leydi, 1851)
Reference	This study	Ribeiro (1941)	Harnoster et al. (2019)	Mascarenhas and Müller (2017)	Ivashkin et al. (1977)	Baylis and Daubney (1922)	Dujardin (1845)	Sharma et al. (2002)	Moravec and Vargas-Vázquez (1998)
Host	*Rhinoclemmys punctularia*	*Podocnemis expansa*	*Rhinoclemmys punctularia*	*Trachemys dorbigni*	*Mauremys reevesi*	*Pangshura smithii*	*Emys orbicularis*	*Cuora amboinensis*	*Trachemys scripta*
Country	Brazil	Brazil	French Guiana	Brazil	China	India	France	Malaysia	Mexico
Number of ridges in valves	♂ 11−18	♂ 17−20	♂ 11−15	♂ 12−17	♂ 10	♂ 8−10	♂ 10	♂ 8−12	♂ 16−21
	♀ 10−17	♀ 17−20	♀ 10−14	♀ 12−17	♀ 10	♀ 8−10	♀ 10	♀ 8−12	♀ 16−21
Pairs of caudal papillae (pre:ad:post)	7:2:3	7:0:2	6:2:5	7:2:4	7:2:5	7:2:6	7:0:5	7:2:5	7:0:6
Total length (mm)	♂ 9.72−11.73	♂ 11.95	♂ 9.71−12.25	♂ 6.55−11.4	♂ 5.65−7.90	♂ 10.9−14.5	♂ 6.93−10.84	♂ 7.45−11.50	♂ 4.43−7.53
	♀ 15.24−18.00	♀ 15.08−17.92	♀ 13.73−18.49	♀ 11.28−18.71	♀ 11.05−12.1	♀ 20.8−22.0	♀ 11.68–12.73	♀ 7.57−21.05	♀ 7.47−10.35
Tail	♂ 157−227	♂ 130	♂ 184−365	♂ –	♂ 550−560	♂ 210	♂ 62	♂ 140−190	♂ 84−87
	♀ 237−544	♀ 210	♀ 215−560	♀ 220−320	♀ 170−210	♀ 330	♀ 172	♀ 100−325	♀ 150−218
Right spicule	800−891	570	360−806	800−950	490−630	970	800	700−863	696−759
Left spicule	373−453	240	208−456	300−390	200−260 mm	430	380	320−370	207−210
Vulva (mm)	7.71−10.81	8.64−9.17	6.98−10.06	5.48–9.61	0.52−0.60	9.1−10.4	5.65	4.02−10.50	3.06−5.02
Vulvar lips morphology	Anterior lip enlarged, well-developed; posterior one indistinct; small lateral projection near the vulvar opening present	Some specimens with a prominent anterior lip, others with a prominent posterior lip	Anterior lip enlarged, well-developed; posterior one indistinct	Anterior lip well-developed; posterior one indistinct	Indistinct lips	Prominent lips, with the anterior one being considerably larger than the posterior, and overlapping it	Anterior lip well-developed; posterior one indistinct	Anterior lip protruding; posterior lip small	Anterior lip protruding; posterior lip small^a^
Phasmids	♂ present	♂ –	♂ –	♂ present	♂ –	♂ –	♂ –	♂ present	♂ –
	♀ present	♀ –	♀ –	♀ present	♀ –	♀ present	♀ –	♀ present	♀ –
Mucrons on the female tail	4	3	3	3	3	2	3	3	3

*Notes*: ^a^Based on Ivashkin et al. (1977).

## Discussion

Following the classification proposed by Silva et al. (2026), the specimens examined in the present study were assigned to *Serpinema*. The unique morphological features of *Serpinema yehi* n. sp., together with the molecular evidence, support its recognition as a new species and further corroborate the validity of *Serpinema* as a distinct genus.

Our phylogenetic analyses revealed that *Se. yehi* n. sp. forms a well-supported clade with *Se. cayennensis* described by Harnoster et al. (2019). Notably, both species parasitize *Rhynoclemmys punctularia* in the Neotropical Region. However, the limited availability of COI sequences for *Serpinema* spp. prevents a more robust assessment of this relationship. Thus, it remains unclear whether this clustering reflects genuine phylogenetic affinity at the genus level or is additionally influenced by shared host association and common occurrence within the Neotropical biogeographical region.

Phylogenetic analyses based on the 18S marker recovered well-supported clades composed of individual species. This pattern aligns with the typically low genetic divergence observed among conspecific individuals, even across populations separated by broad geographic distances. Notably, *Sp. inopinatus* formed a well-supported subclade containing three sequences from different regions: two derived from specimens parasitizing freshwater fishes in Brazil and one from a marine fish from Egypt.

However, this subclade nested within a broader, well-supported clade comprising *Sp. hilarii* and *Sp. pintoi*, both of which parasitize South American freshwater fishes. This grouping may reflect the influence of host habitat and biogeographical distribution on parasite phylogeny, as suggested by Ailán-Choke and Pereira (2021).

The well-supported clade containing *Pr. laeviconchus* and *Pr. pseudolaeviconchus*, both from the African continent, further supports the hypothesis that host geographical distribution may influence parasite phylogeny. Nofal et al. (2022) proposed that sequence KP274849 (attributed to *Pr. laeviconchus*) actually represents *Pr. pseudolaeviconchus*, based on its substantial genetic divergence from sequence JF803934 (3% genetic divergence in our study), the only other sequence available at the time for *Pr. laeviconchus*. However, KP274849 (*Pr. laeviconchus*) also exhibited marked divergence from sequences attributed to *Pr. pseudolaeviconchus* in their analyses (5% genetic divergence in our study), leading the authors to conclude that the relationship between these two nominal species requires further investigation.

In our phylogenetic reconstruction, each nominal species formed distinct subclades. However, the three sequences assigned to *Pr. laeviconchus* (KP274849, PP581381 and JF803934) showed high intraspecific genetic divergence (2% between KP274849 and PP581381; 3% between KP274849 and JF803934; and 5% between PP581381 and JF803934). Our findings suggest that these sequences may not represent a single taxon, or alternatively, that *Pr. laeviconchus* constitutes a cryptic species, distinguishable only through molecular approaches.

*Denticamallanus rarus* (DQ494195) and *Pr. pacificus* (DQ442665) clustered in a well-supported clade with 0% genetic divergence, suggesting that they represent the same taxon. Conversely, the two currently available sequences for *D. rarus* (DQ494195 and JF803912) showed substantial genetic divergence (6%) and did not cluster together, indicating a probable case of morphological misidentification, as previously suggested by Nofal et al. (2022).

This discrepancy may result from errors during sequence submission or from experimental contamination, particularly because sequences DQ494195 and DQ442665 originated from the same study (Wijová et al., 2006). We conclude that sequence DQ494195, originally attributed to *D. rarus*, corresponds to the species *Pr. pacificus*.

The remaining sequence of *D. rarus* (JF803912) clustered in a well-supported clade with *Sp. huacraensis*. Notably, both taxa share the presence of tooth-like structures in the buccal capsule (Ailán-Choke et al., 2019; Ailán-Choke and Pereira, 2021). Based on this shared morphology and the monophyletic clade recovered both here and by Ailán-Choke and Pereira (2021), this provides additional evidence to the conclusions made by Silva et al. (2026), who proposed that *Sp. huacraensis* should be reassigned to *Denticamallanus*. Our findings also support the recognition of *Denticamallanus* as a valid and distinct genus. We emphasize that tooth-like structures represent a reliable morphological synapomorphy for the group.

In the 18S phylogenetic reconstruction, *Se. yehi* n. sp. did not cluster with any other available sequence. Instead, it formed a well-supported clade with *C. pallidipectoris* (1% of genetic divergence) and *Zeylanema* sp. (1% of genetic divergence). The proximity between the new species and *C. pallidipectoris* highlights the influence of host phylogeny in the evolution of these nematodes, as both species parasitize freshwater turtles.

Furthermore, *C. pallidipectoris* did not group with other *Camallanus* species. This suggests that chelonian-parasitizing *Camallanus* are more closely related to *Serpinema* than among *Camallanus* spp. infecting other host groups. Although *Zeylanema* sp. parasitizes a fish host and resembles *Camallanus* morphologically, its sequence did not group with fish-parasitizing *Camallanus* species.

Based on 28S ribosomal DNA, Saito et al. (2025) also recovered a clade comprising *Camallanus, Serpinema* and *Zeylanema*, leading them to propose that *Serpinema* and *Zeylanema* might be treated as junior synonyms or subgenera of *Camallanus*. However, Ailán-Choke and Pereira (2021) and Silva et al. (2026) rejected the use of subgeneric classification within Camallanidae. Therefore, a more robust assessment of the taxonomic status of *Zeylanema* will require the inclusion of additional sequences from its representatives.

Consistent with 18S-based analyses, the COI phylogeny recovered several well-supported single-species clades. *Serpinema yehi* n. sp. clustered with *Se. cayennensis* (6% of genetic divergence) in a strongly supported clade, which nested outside the subclade comprising *Camallanus* spp. Although *Camallanus, Serpinema* and *Zeylanema* formed a single major clade, with *Camallanus* and *Serpinema* forming distinct subclades, *Zeylanema* grouped with *Serpinema*.

This topology reinforces the close phylogenetic relationships among these three genera, suggesting relatively recent divergences. The observed evolutionary proximity aligns with their marked morphological similarity, as, according to Silva et al. (2026), these taxa are distinguished by the morphology and arrangement of the internal ridges of the buccal capsule. Additionally, *C. kaapstaadi* and *C. xenopodis*, both parasites of *Xenopus laevis* from South Africa, formed a distinct subclade separate from other *Camallanus* species, further confirming that the genus *Camallanus* is not monophyletic.

Our results support the classification by Silva et al. (2026), which rejects the subgeneric classification within Camallanidae and recognizes *Camallanus, Serpinema* and *Zeylanema* as distinct genera. However, a comprehensive understanding of the phylogenetic relationships among these three taxa will require additional molecular data, particularly from underrepresented taxa of *Zeylanema* and *Serpinema*.

*Spirocamallanus xenopodis* (MN523681) and *Pr. slomei* (MG948463), both parasitizing anurans of the genus *Xenopus* in South Africa and previously classified in *Batrachocamallanus*, clustered in a fully supported clade. Notably, the two COI sequences are nearly identical (99.85% identical) and show 0% genetic divergence, suggesting that they may represent the same taxon, as Nofal et al. (2022) previously observed. However, Svitin et al. (2019) reported a 2.9% divergence between *Sp. xenopodis* and *Pr. slomei* based on 28S sequences. Given this discrepancy, we agree with Nofal et al. (2022) that a sequence submission error likely explains the absence of genetic divergence among COI sequences, rather than biological conspecificity.

Traditional taxonomy uses buccal capsule morphology as a key diagnostic character for camallanid subfamilies and genera; however, molecular evidence suggests that this trait does not necessarily correspond to monophyletic lineages (Ailán-Choke and Pereira, 2021). Both our COI and 18S phylogenetic analyses clearly recovered this pattern. Consequently, the subdivision of Camallanidae into subfamilies based on buccal capsule morphology appears to be entirely artificial, as our results failed to recover exclusive clades for either Procamallaninae or Camallaninae.

Our analyses provide evidence for the paraphyly of Camallanidae, demonstrating that distinct buccal capsule morphologies arose independently throughout the family’s evolutionary history. Both the smooth internal-surface buccal capsule, typical of *Procamallanus*, and the spiral ridges, characteristic of *Spirocamallanus*, appear to have originated multiple times during camallanid diversification. These results align with 28S-based phylogenetic analyses by Svitin et al. (2019), which recovered species with smooth internal capsules and spiral ridges within the same clade.

The lack of well-supported clades restricted to *Procamallanus* and *Spirocamallanus* demonstrates the limited taxonomic utility of buccal capsule internal ridges for generic delimitation within the subfamily Procamallaninae. While these characters remain useful for morphological identification and systematic arrangement, they do not accurately reflect the phylogenetic relationships or the evolutionary processes that occurred during the diversification of these nematodes. Nevertheless, for the practical purposes of systematic organization, we follow the classification proposed by Silva et al. (2026).

Our phylogenetic study corroborates the three factors proposed by Ailán-Choke and Pereira (2021) as primary drivers of camallanid diversification and evolution: host phylogeny, habitat and biogeography. For instance, the clade comprising *Sp. barlowi* and *Sp. rebecae*, both parasites of freshwater fishes from the Nearctic and Neotropical regions (geographically close areas), illustrates this pattern. Similarly, in COI phylogeny, the clade formed by *C. cotti* and *C. hypophthalmichthys*, both originating from Asia, reinforces the roles of geographic distribution and habitat in structuring the phylogeny of Camallanidae.

However, this pattern was not consistent across all taxa. In our COI analyses, the *Sp. inopinatus* clade (Brazilian freshwater parasites) did not cluster with either *Sp. barlowi*/*Sp. rebecae* group or the *Sp. neocaballeroi* clade, despite their shared freshwater habitats and Nearctic/Neotropical distributions. This discrepancy suggests that, while biogeography and habitat are important, additional factors, such as host evolutionary history or species-specific ecological traits, also play a significant role in shaping the phylogeny of Camallanidae.

Interestingly, although *C. kaapstaadi, C. xenopodis* (Camallaninae), *Pr. slomei* and *Sp. xenopodis* (Procamallaninae) all parasitize South African amphibians of the genus *Xenopus* (Svitin et al., 2018, 2019), they did not group within the same subclade in our COI analyses. Instead, the two *Camallanus* species clustered together, whereas *Pr. slomei* and *Sp. xenopodis* formed a separate, distinct clade. This topology suggests a clear phylogenetic separation between the Camallaninae and Procamallaninae nematode lineages in this host group, despite their shared host and biogeographic affinities.

In the 18S phylogenetic tree, *Sp. inopinatus, Sp. hilarii* and *Sp. pintoi* formed a well-supported clade with parasites of freshwater fishes from the Neotropics. However, a single sequence of *Sp. inopinatus* from a marine host in Egypt clustered within this group, showing 0% genetic divergence from three sequences of *Sp. inopinatus*. This finding suggests that, although biogeography shapes camallanids evolution, conspecific sequences still tend to cluster together, regardless of whether hosts are from geographically distant regions or occupying distinct habitats.

The COI-based phylogenetic tree for the subfamily Camallaninae resulted in three well-supported clades: one corresponding to *Pa. cyathopharynx* (also recovered in the other two phylogenetic analyses); a second clade representing the genus *Serpinema*, including *Se. yehi* n. sp. and *Se. cayennensis*; and a third clade corresponding to *C. cotti*. However, this reconstruction placed *Serpinema* and *Paracamallanus* in the same subclade, and the *C. cotti* clade did not cluster with *C. hypophthalmichthys*, contradicting the topology of 18S and the broader COI tree. These inconsistencies further confirm that *Camallanus* is a non-monophyletic group. Additionally, the unstable position of this taxon reinforces the need for additional *Zeylanema* sequences to establish a robust taxonomic status of this genus.

*Serpinema yehi* n. sp. represents the eighth species of the genus recorded in Brazil and the second species described parasitizing the freshwater turtle *R. punctularia*, the spot-legged turtle. This turtle species, commonly known as ‘perema’, is distributed throughout the Brazilian Amazon and is semiaquatic and diurnal. It is omnivorous, feeding primarily on fruit but also consuming animal-derived items (Ferrara et al., 2017).

Silva et al. (2023) suggested that turtle feeding habits strongly influence exposure to different nematode larvae in aquatic environments, through the consumption of small prey, such as crustaceans, amphibians and fishes, which may harbour camallanid larvae. This dietary behaviour may contribute to the high diversity of *Serpinema* species reported from a single host, such as *K. scorpioides* (Mascarenhas and Müller, 2021; Silva et al., 2023; Conga et al., 2026). In contrast, the more restricted diet of *R. punctularia* may be associated with the comparatively lower diversity of *Serpinema* spp. recorded from this host, particularly relative to other freshwater turtles.

Our results support the description of a new species of *Serpinema* based on both morphological and molecular evidence, as well as the recognition of *Serpinema* as a valid genus distinct from *Camallanus*. Additionally, they reinforce the polyphyly of *Camallanus* and the artificial nature of traditional subfamily divisions. Thus, the independent origin of internal buccal ridges limits their phylogenetic signal and delimitation of monophyletic lineages. However, despite the evolutionary homoplasy in buccal structures, these structures remain indispensable for practical systematic organization and morphological identification of Camallanidae.

Host phylogeny, habitat and biogeography significantly shape camallanid diversification and evolution. However, the genetic stability of *Sp. inopinatus* across different regions suggests that species identity remains conserved despite extreme geographic or environmental shifts. Additionally, the unstable position of certain clades and the identification of likely database errors underscore the urgent need for expanded molecular sampling, particularly for the genus *Zeylanema*, to establish robust evidence to understand the evolutionary processes for the family.

## Supporting information

10.1017/S0031182026102509.sm001 Silva et al. supplementary material 1 Silva et al. supplementary material

10.1017/S0031182026102509.sm002 Silva et al. supplementary material 2 Silva et al. supplementary material

10.1017/S0031182026102509.sm003 Silva et al. supplementary material 3 Silva et al. supplementary material

## Supplementary material

The supplementary material for this article can be found at https://doi.org/10.1017/S0031182026102509.

## Table 1 Long description

Camallanid nematode species are listed by subfamily, with updated species names alongside the corresponding NCBI database name, host, country of record, habitat, and available gene sequences used for phylogenetic analyses. Most taxa fall under Camallaninae and Procamallaninae, plus three outgroup species. Records cover a wide geographic range including South Africa, China, Mexico, Argentina, Brazil, Peru, New Caledonia, India, and other locations, and include both freshwater and marine habitats, with freshwater appearing more frequently. Many species have multiple rows reflecting different hosts, countries, or sequence submissions, such as Camallanus cotti, Procamallanus laeviconchus, and several Spirocamallanus species. Sequence availability is uneven: 18S accessions are common, while COI is missing for many entries; a smaller subset provides both markers, for example Paracamallanus cyathhopharynx, Procamallanus pseudolaeviconchus, Procamallanus spiculogubernaculus, Zeylanema sp., and some Spirocamallanus records. Several entries note unpublished sequences or incomplete metadata such as missing country, habitat, or host identification, which limits direct comparisons across all rows. The table also highlights naming mismatches where the study’s updated taxonomy differs from the NCBI name used for deposited sequences, especially among Procamallanus and Spirocamallanus listings.

Navigate back to Table 1.

## Table 2 Long description

Thirteen Serpinema nematode species are grouped into two morphogroups and compared by host turtle, country of record, literature reference, and key morphology and measurements for males and females. For each species, the table lists ridge counts, caudal papillae pattern, total body length, tail length, spicule length (single spicule in morphogroup I; right and left spicules in morphogroup II), vulva position, vulvar lip shape, phasmid presence, and number of mucrons on the female tail. Morphogroup I includes four species from India and Brazil, mostly with about nine to sixteen ridges and female mucrons typically three when reported; one species lacks spicule and vulva values in the source. Morphogroup II includes nine species spanning Brazil, French Guiana, China, India, France, Malaysia, and Mexico, with ridge counts ranging from about eight up to the low twenties depending on species. Across morphogroup II, right spicules are consistently longer than left spicules, and several species report phasmids present in males and females while others have no report. Body size varies widely: some species have males under eight millimeters while others exceed ten millimeters, and females commonly exceed males in total length. Vulvar lip descriptions differ by species, ranging from indistinct lips to prominent anterior lips that may overlap the posterior lip, which can help separate otherwise similar taxa. Values are compiled from multiple references and some cells are missing or marked as not reported, so comparisons should be interpreted as literature-based rather than standardized measurements.

Navigate back to Table 2.

## Figure 4 Long description

The diagram is a maximum likelihood phylogenetic tree of Camallanidae species based on the 18S gene. The tree is oriented vertically with branches extending from top to bottom. The outgroup is Spirocerca lupi, labeled at the bottom. The tree is divided into two main clades, Camallaninae and Camallaninae, highlighted with blue shading. Each branch is labeled with species names and GenBank accession numbers. Node support values are shown above or below branches, with some values omitted or indicated by a dash if below thresholds. The position of Serpinema yehi n. sp. is marked in bold italics. The branch-length scale bar at the bottom left represents the number of substitutions per site, set at 0.03. The tree illustrates the evolutionary relationships among the species, with specific nodes indicating divergence points.

Navigate back to Figure 4.

## Figure 5 Long description

A horizontal phylogenetic tree is arranged left to right, with branching lines extending from a left trunk toward taxon labels on the right. Two wide horizontal shaded bands on the left are each labeled, Camallaninae. At the bottom, a separate baseline is labeled, Outgroup. A scale bar at the lower left is labeled, 0.4. The two outgroup tips at the lower right read, Ancyracanthus electrophoris OP765585 and Spiroxys ankarafantsika MW545829. Within the main tree, many tip labels show a genus and species name followed by an accession code. Near the middle right, the label, Serpinema yehi n. sp. ZJ113265 appears in bold italics and is paired as a two tip split with, Serpinema cayennensis MN104841. A node label next to the branch leading to this Serpinema pair reads, 0.90 slash 98. Other node labels appear at multiple branch points as numbers or as a dash, including 1 slash 100 near the left trunk and 0.99 slash 98 near the upper right. Several nearby tips include Camallanus cotti with multiple accession codes, and other genera including Spirocamallanus, Paracamallanus, and Procamallanus, each shown as clusters of branching tips.

Navigate back to Figure 5.

## Figure 6 Long description

The diagram is a maximum likelihood phylogenetic tree of species within the subfamily Camallaninae, based on the COI gene. The tree uses Spiroxys ankarafantsika and Ancyracanthus electrophoris as outgroups. The main branch labeled 'Camallaninae' splits into several sub-branches representing different species, each with GenBank accession numbers. Camallanus kaapstaadi MG948461, Zeylanema sp. LC815080, and Camallanus xenopodis MG948462 form one clade. Another clade includes Paracamallanus cyathopharynx with accession numbers OP602981, OP602980, and MN523683. Serpinema cayennensis MN104841 and Serpinema yehi n. sp. PZ113265 form a separate clade. Camallanus cotti EU598890 and EU598886, along with Camallanus hypophthalmichthys EU598816, are grouped together. Node support values are shown above or below branches, with posterior probabilities and bootstrap values indicated. The branch-length scale bar represents the number of substitutions per site, marked as 0.2. The position of Serpinema yehi n. sp. is highlighted in bold italics.

Navigate back to Figure 6.
